# MRI in congenital duplication of urethra

**DOI:** 10.4103/0971-3026.54884

**Published:** 2009-08

**Authors:** S Bhadury, Umesh C Parashari, Ragini Singh, Neera Kohli

**Affiliations:** Department of Radiodiagnosis, C.S.M. Medical University (formerly King George Medical University), Lucknow, Uttar Pradesh, India

**Keywords:** Congenital, MRI, urethral duplication

## Abstract

Congenital urethral duplication is a rare anomaly, with less than 200 cases described in the literature. The investigations that are usually performed are micturating cystourethrography (MCU) and retrograde urethrography (RGU), which can diagnose the presence of duplication but cannot diagnose the precise relationship of the duplicated urethra with other pelvic structures. MRI, because of the excellent tissue contrast that it provides and its multiplanar ability, can demonstrate with precision, the size, shape and position of the two urethras. We describe below a case where MRI was able to show this exquisitely.

## Introduction

Congenital urethral duplication is a rare congenital anomaly, with less than 200 cases described in the literature.[1,2] The investigations usually performed in cases of urethral duplication are voiding cystourethrography and distal urethrography (RGU); these investigations can diagnose whether there is a duplication or not but cannot diagnose the precise relation of the duplicated urethras with other pelvic structures. MRI, with its superior contrast resolution and multiplanar capability is usually able to delineate the exact urethral anatomy much better.

## Case Report

A 5-year-old child was brought with the complaint of passage of urine through the anal opening since birth.

**Figure 1:**
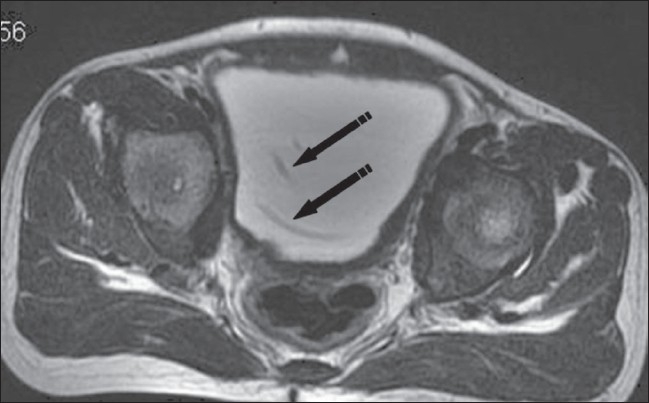
Axial T2W image shows two catheters (arrows) within the urinary bladder lumen

**Figure 2 (A, B):**
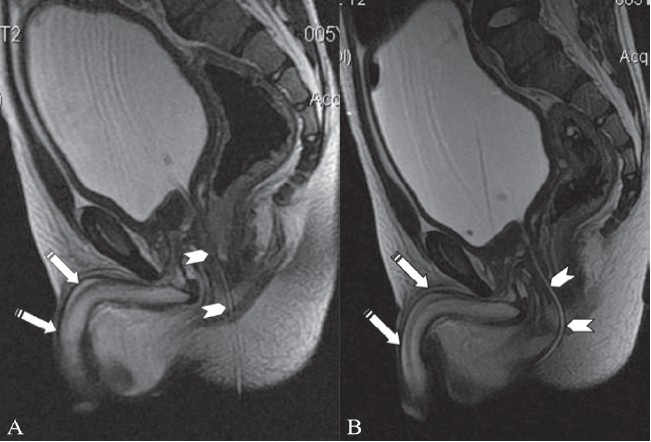
Sagittal T2W images show a small caliber cannula in the anterior urethra (arrows), passing through the penis (linear hypointense structure in the penis) with the posterior urethra (arrowhead) passing through the rectum (type IIA urethral duplication)

**Figure 3:**
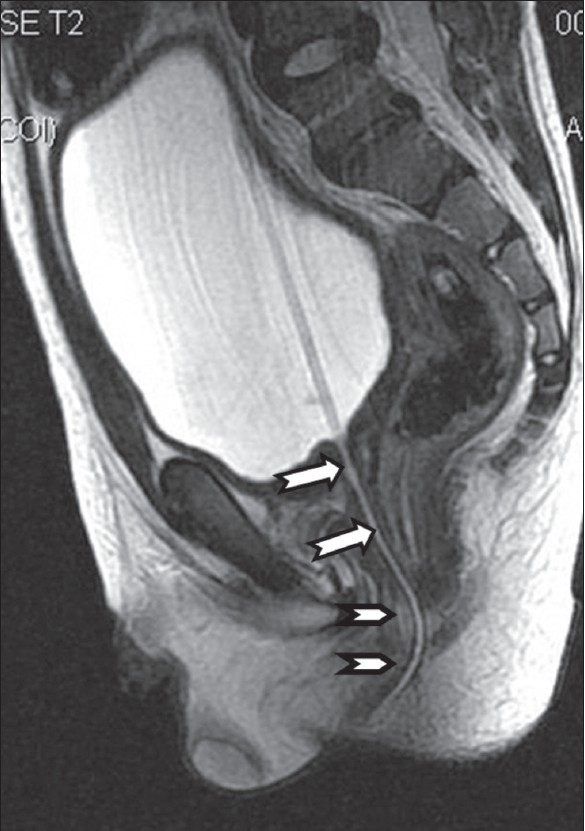
Sagittal T2W image shows the posterior urethra (arrow) passing through the anal canal (arrowhead) with the catheter *in situ*

**Figure 4:**
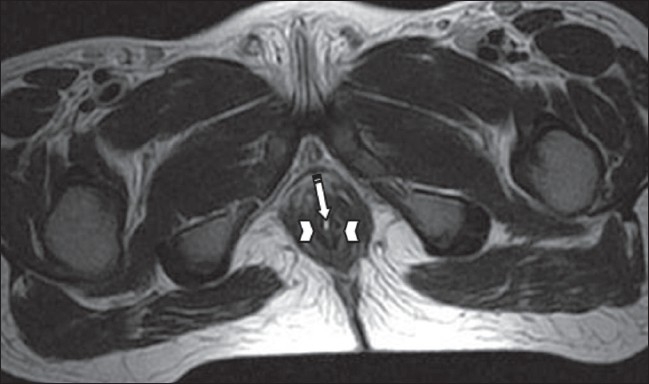
Axial T2W image shows the posterior catheter (arrow) coming out through the anal canal (arrowheads)

The patient was suspected to have two bladder outlet routes, and MRI was performed to detect the course of the two urethras and the position of the prostate. The bladder was first filled with gadolinium and then the two suspected urethras were cannulated using thin (no. 8) infant feeding tubes. The purpose of cannulation was to properly demonstrate the outline of the posterior urethra, which was not demonstrated well without cannulation. MRI was performed while continuously injecting saline into the cannulas. The study showed the two catheters within the urinary bladder, one entering anteriorly and the other entering the posterior aspect of the bladder [Figure 1]. The anterior catheter was seen to course along the penis and this urethra was found to be of a smaller caliber. The prostate was seen encircling the posteriorly located urethra [Figure 2]. The second catheter was seen to exit posteriorly, opening into the anterior wall of the anal canal 2.3 cm from the external anal sphincter [Figures 3 and 4]. The seminal vesicles and ejaculatory ducts of both sides were visualized and were normal. The soft tissues and the bones forming the pelvis were also normal.

## Discussion

Urethral duplication is a rare congenital anomaly; there are less than 200 cases described in the literature.[1] A few cases of urethral duplication have been reported recently.[2,3] This congenital anomaly is characterized by two urethras, which may be either partial or complete. One urethra is usually normal and the other is an accessory urethra.

Currently, the most widely accepted theory for the cause of complete urethral duplication is that of Patten and Barry. According to them, an abnormal relationship exists between the lateral anlagen of the genital tubercle and the ventral end of the cloacal membrane. Normally, these anlagen fuse in front of the genital part of the cloacal membrane, thereby preventing the membrane from extending further ventrally. If the tubercles lie more posteriorly, or the membrane extends more ventrally than usual, a part of the membrane remains in front of the tubercle and interferes with its subsequent growth, thereby causing the lesion.

The forms of urethral duplications are completely different in males and females. In males, urethral duplication is classified into three types (Effman's classification):[4]

Type I: blind-ending accessory urethra (incomplete urethral duplication)

IA. Distal—duplicated urethras opening on the dorsal or ventral surface of the penis but not communicating with the urethra or bladder (the most common type)

IB. Proximal—accessory urethra opening from the urethral channel but ending blindly in the periurethral tissues (rare)

Type II: completely patent accessory urethra. It is divided into two parts: A (two meatuses) and B (one meatus)

IIA1 Two noncommunicating urethras arising independently from the bladder

IIA2 Second channel arising from the first and coursing independently into a second meatus (Y-type)

IIB Two urethras arising from the bladder or posterior urethra and uniting into a common channel distally

Type III: accessory urethras arising from duplicated or septated bladders.

According to this classification, our case is a type IIA urethral duplication.

Accessory urethras may be present dorsal to, ventral to or next to the normal urethra. Rarely, accessory urethras may communicate with the prostatic ducts and seminal vesicles. When the accessory urethra is in the dorsal position, the external meatus can be epispadiac; if in the ventral position, the accessory urethra is usually hypospadiac, and the external urethra can open anywhere from the glans to the penoscrotal junction. The latter may resemble a congenital urethra–perineal fistula[5,6]. In some patients, the accessory urethra may open into the anal canal or at the anorectal junction; this anomaly is referred to as an H-type fistula rather than an accessory urethra. Rarely, when there is no cutaneous or rectal communication, the accessory ventral urethra may form a cyst.

The two duplicated urethras may lie side by side; in these cases, the duplication may be limited to the prostatic urethra. Urethral duplications can also occur with glandular or complete diphallus. The anomalies associated with urethral duplication are superior vesical fissure, bladder exstrophy, posterior urethral valves, imperforate anus, congenital urethral polyps and megalourethra of both channels.

In females, urethral duplication is classified into the following types:

1. Double urethra and double bladder
2. Double urethra, single bladder
3. Accessory urethra posterior to the normal channel
4. Double proximal urethra and single distal urethra
5. Single proximal urethra and duplicated distal urethra.

Trifurcation of urethra has also been reported, but this form exists only in males. It may be associated with congenital unilateral absence of a kidney and testis.

Most patients have no symptoms except for, perhaps, a double stream. Other presentations may be incontinence, urinary tract infections and bladder outflow tract obstruction.

There are a variety of radiological or endoscopic procedures that can be used to define the anatomy of the urethra. These include micturating cystourethrogram (MCUG), intravenous urography, sonourethrography, nuclear scintigraphy and cystourethroscopy.

Voiding cystourethrography and retrograde urethrography should be carried out in lateral projections for visualization of the size, shape and position of the two channels. Effman *et al*. showed that catheterization of a ventrally placed urethra was easier.[4]

IVU may demonstrate a wide symphysis pubis in case of epispadiac accessory urethra. Other associated anomalies are unilateral renal agenesis, ureteral duplication and a duplicated bladder.

USG can demonstrate the exact length of any stricture segment or any extraluminal abnormal soft tissue or diverticulations. However, USG is user-dependent. The advantage is the lack of radiation exposure.

MRI is an excellent investigation for the evaluation of duplicated urethras and the periurethral soft tissues. MRI can demonstrate with precision the sizes, shapes and positions of the two urethras as well as other associated genitourinary abnormalities. However, till recently MRI has only been used as an adjunct for the visualization of the urethra.[^7^]

Indications for surgery include annoying symptoms such as a double stream, urinary incontinence, urinary obstruction or associated genitourinary anomalies. In our case, the patient underwent surgical correction and was normal at 3 months' follow-up.

Differential diagnoses include lacuna magna (which is a problem especially in patients with hypospadias, when the lacuna lies distal to the meatus), acquired fistulous tracts (postinfective and post-instrumentation), urethral diverticula and dilated Cowper's gland.
